# Poly[dichloridobis(μ_2_-di-4-pyridyl sulfide-κ^2^ *N*,*N*′)cobalt(II)]

**DOI:** 10.1107/S1600536809001676

**Published:** 2009-02-04

**Authors:** Jian-Ge Wang, Jian-Hua Qin, Gui-Ying Zhang

**Affiliations:** a College of Chemistry and Chemical Engineering, Luoyang Normal University, Luoyang 471022, People’s Republic of China

## Abstract

In the title compound, [CoCl_2_(C_10_H_8_N_2_S)_2_]_*n*_, the Co^II^ atom is located on an inversion centre and is six-coordinated by four N atoms of four symmetry-related di-4-pyridyl sulfide ligands, and two Cl atoms in *trans* positions, in a distorted octa­hedral geometry. The bridging bidentate di-4-pyridyl sulfide ligands link the Co^II^ centres into a three-dimensional network. The four coordinating pyridine groups are donors and acceptors (N atoms) for intra­molecular C—H⋯N and C—H⋯Cl hydrogen bonds.

## Related literature

For di-4-pyridyl sulfide metal complexes, see: Jung *et al.* (1998 ▶, 1999 ▶); Kondo *et al.* (2004 ▶); Muthu *et al.* (2005 ▶). 
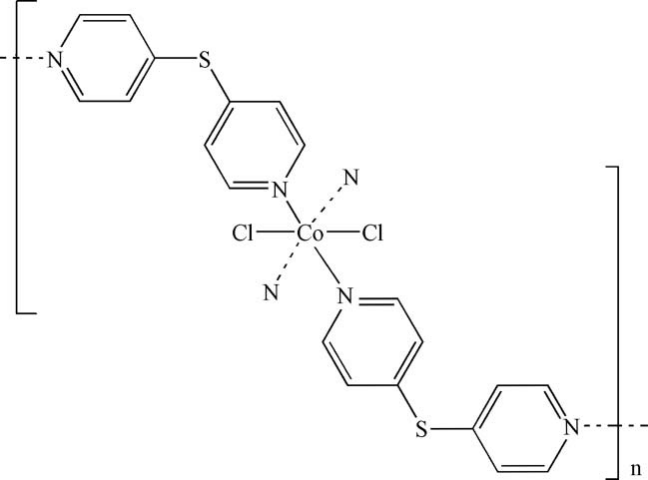


## Experimental

### 

#### Crystal data

[CoCl_2_(C_10_H_8_N_2_S)_2_]
*M* *_r_* = 506.32
Monoclinic, 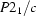

*a* = 7.4940 (11) Å
*b* = 15.355 (2) Å
*c* = 9.4009 (14) Å
β = 98.413 (2)°
*V* = 1070.1 (3) Å^3^
*Z* = 2
Mo *K*α radiation
μ = 1.26 mm^−1^
*T* = 296 (2) K
0.44 × 0.34 × 0.24 mm

#### Data collection

Bruker SMART CCD area-detector diffractometer
Absorption correction: multi-scan (*SADABS*; Bruker, 1997 ▶) *T*_min_ = 0.608, *T*_max_ = 0.747
5174 measured reflections
1969 independent reflections
1720 reflections with *I* > 2σ(*I*)
*R*_int_ = 0.017

#### Refinement

*R*[*F* ^2^ > 2σ(*F* ^2^)] = 0.027
*wR*(*F* ^2^) = 0.070
*S* = 1.05
1969 reflections
133 parameters
H-atom parameters constrained
Δρ_max_ = 0.38 e Å^−3^
Δρ_min_ = −0.26 e Å^−3^

### 

Data collection: *SMART* (Bruker, 1997 ▶); cell refinement: *SAINT* (Bruker, 1997 ▶); data reduction: *SAINT*; program(s) used to solve structure: *SHELXS97* (Sheldrick, 2008 ▶); program(s) used to refine structure: *SHELXL97* (Sheldrick, 2008 ▶); molecular graphics: *SHELXTL* (Sheldrick, 2008 ▶); software used to prepare material for publication: *SHELXTL*.

## Supplementary Material

Crystal structure: contains datablocks I, global. DOI: 10.1107/S1600536809001676/si2149sup1.cif

Structure factors: contains datablocks I. DOI: 10.1107/S1600536809001676/si2149Isup2.hkl

Additional supplementary materials:  crystallographic information; 3D view; checkCIF report

## Figures and Tables

**Table:** 

Co1—N1	2.2185 (18)
Co1—N2^i^	2.2822 (17)
Co1—Cl1	2.4221 (5)

**Table:** 

N1—Co1—N2^i^	94.00 (6)
N1—Co1—Cl1	90.50 (5)

Symmetry code: (i) 
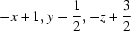
.

**Table 2:** Hydrogen-bond geometry (Å, °)

*D*—H⋯*A*	*D*—H	H⋯*A*	*D*⋯*A*	*D*—H⋯*A*
C5—H5⋯N2^ii^	0.93	2.62	3.119 (3)	114
C6—H6⋯Cl1^iii^	0.93	2.66	3.292 (2)	126
C10—H10⋯Cl1^iv^	0.93	2.64	3.292 (2)	128

Symmetry codes: (ii) 
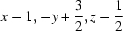
; (iii) 
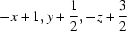
; (iv) 
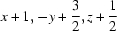
.

**Fig. 1.**
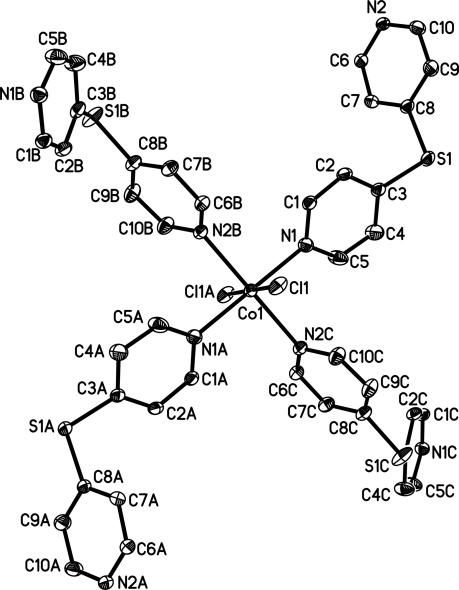
A view of the local coordination of the Co(II) cation in the title compound. Displacement ellipsoids are drawn at the 30% probability level. Symmetry codes: (A) (-x, 1 - y, 1 - z); (B) (-1 + x, 3/2 - y, -1/2 + z); (C) (1 - x, -1/2 + y, 3/2 - z).

**Fig. 2.**
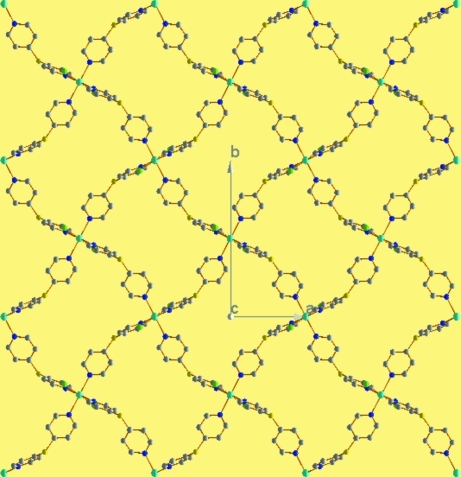
A view of the two-dimensional network.

**Fig. 3.**
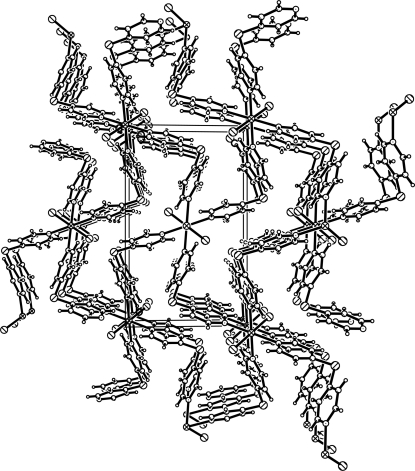
A view of the compound packing down the a axis.

## supplementary crystallographic information

### Comment

As well known, di-4-pyridyl sulfide possesses a magic angle (C-S-C, ~100°) and conformational nonrigidity so it has some flexibility compared with other linear rigid ligands such as simple 4, 4'-bipyridine analogues. A number of metal complexes derived from di-4-pyridyl sulfide have been reported previously, such as the silver(I) complexes (Jung *et al.,* 1999), copper(II) complexes (Muthu *et al.,* 2005), nickel(II) complex (Kondo *et al.,* 2004), as well as the cobalt(II) complex that showing 2-fold interpenetrating structures (Jung *et al.,* 1998).

As shown in Fig. 1, the local geometry of the cobalt atoms is a distorted octahedral arrangement with two chlorine atoms in trans positions and four pyridine units in a propeller arrangement (Tab. 1). Each di-4-pyridyl sulfide ligand connects two cobalt(II) ions defining the edges of a 40-membered [Co(II)]_4_ sheet (Fig. 2). The bent angle of the sulfur atom [C-S-C = 102.90 (10) °]. The Co-Co separation through a di-4-pyridyl sulfide ligand is 11.2646 (10) Å, and through the diagonal of the rhombus is 15.355 (2) Å. There are six intramolecular C—H···N and C—H···Cl hydrogen bonding contacts around the coordination sphere of the cobalt atom (Tab. 2). The packing of the layered structure is shown in Fig.3.

### Experimental

To a stirred solution of di-4-pyridyl sulfide (0.5 mmol) in ethanol-H_2_O 20 ml (v/v, 1:1) was added solid CoCl_2_(0.5 mmol). Then the obtained mixture was basified with NaOH (0.5 mol/l) to a pH of 6.0 and stirred at 343K for 4h, filtrated. One week later, red crystals appeared.

### Refinement

The H atoms were positioned geometrically and treated as riding, with C—H = 0.93 Å (CH) and *U*ĩso~(H) = 1.2Ueq(C).

### Crystal data

**Table:**

[CoCl_2_(C_10_H_8_N_2_S)_2_]	*F*(000) = 514
*M**_r_* = 506.32	*D*_x_ = 1.571 Mg m^−^^3^
Monoclinic, *P*2_1_/*c*	Mo *K*α radiation, λ = 0.71073 Å
Hall symbol: -P 2ybc	Cell parameters from 2498 reflections
*a* = 7.4940 (11) Å	θ = 2.6–29.0°
*b* = 15.355 (2) Å	µ = 1.26 mm^−^^1^
*c* = 9.4009 (14) Å	*T* = 296 K
β = 98.413 (2)°	Block, red
*V* = 1070.1 (3) Å^3^	0.44 × 0.34 × 0.24 mm
*Z* = 2	

### Data collection

**Table:**

Bruker SMART CCD area-detector diffractometer	1969 independent reflections
Radiation source: fine-focus sealed tube	1720 reflections with *I* > 2σ(*I*)
graphite	*R*_int_ = 0.017
φ and ω scans	θ_max_ = 25.5°, θ_min_ = 2.6°
Absorption correction: multi-scan (*SADABS*; Bruker, 1997)	*h* = −9→8
*T*_min_ = 0.608, *T*_max_ = 0.747	*k* = −14→18
5174 measured reflections	*l* = −11→11

### Refinement

**Table:**

Refinement on *F*^2^	Primary atom site location: structure-invariant direct methods
Least-squares matrix: full	Secondary atom site location: difference Fourier map
*R*[*F*^2^ > 2σ(*F*^2^)] = 0.027	Hydrogen site location: inferred from neighbouring sites
*wR*(*F*^2^) = 0.070	H-atom parameters constrained
*S* = 1.05	*w* = 1/[σ^2^(*F*_o_^2^) + (0.0329*P*)^2^ + 0.5314*P*] where *P* = (*F*_o_^2^ + 2*F*_c_^2^)/3
1969 reflections	(Δ/σ)_max_ < 0.001
133 parameters	Δρ_max_ = 0.38 e Å^−^^3^
0 restraints	Δρ_min_ = −0.26 e Å^−^^3^

### Fractional atomic coordinates and isotropic or equivalent isotropic displacement parameters (Å^2^)

**Table:**

	*x*	*y*	*z*	*U*_iso_*/*U*_eq_	
Co1	0.0000	0.5000	0.5000	0.02605 (13)	
Cl1	0.20061 (7)	0.43179 (4)	0.35327 (6)	0.04042 (16)	
S1	0.46119 (10)	0.86590 (4)	0.38777 (6)	0.0496 (2)	
N1	0.1375 (2)	0.62685 (11)	0.48432 (19)	0.0336 (4)	
N2	0.8140 (2)	0.95816 (11)	0.79735 (18)	0.0295 (4)	
C1	0.3135 (3)	0.63405 (14)	0.5315 (2)	0.0319 (5)	
H1	0.3717	0.5866	0.5793	0.038*	
C2	0.4148 (3)	0.70682 (14)	0.5143 (2)	0.0335 (5)	
H2	0.5363	0.7088	0.5527	0.040*	
C3	0.3335 (3)	0.77696 (14)	0.4393 (2)	0.0336 (5)	
C4	0.1498 (3)	0.77243 (16)	0.3907 (3)	0.0485 (6)	
H4	0.0894	0.8187	0.3410	0.058*	
C5	0.0589 (3)	0.69710 (16)	0.4181 (3)	0.0492 (6)	
H5	−0.0651	0.6952	0.3886	0.059*	
C6	0.6455 (3)	0.92970 (14)	0.8003 (2)	0.0316 (5)	
H6	0.6015	0.9310	0.8877	0.038*	
C7	0.5337 (3)	0.89872 (14)	0.6820 (2)	0.0349 (5)	
H7	0.4174	0.8803	0.6902	0.042*	
C8	0.5962 (3)	0.89534 (14)	0.5507 (2)	0.0335 (5)	
C9	0.7705 (3)	0.92370 (16)	0.5454 (2)	0.0433 (6)	
H9	0.8182	0.9221	0.4595	0.052*	
C10	0.8725 (3)	0.95443 (16)	0.6695 (2)	0.0411 (6)	
H10	0.9889	0.9737	0.6640	0.049*	

### Atomic displacement parameters (Å^2^)

**Table:**

	*U*^11^	*U*^22^	*U*^33^	*U*^12^	*U*^13^	*U*^23^
Co1	0.0256 (2)	0.0273 (2)	0.0247 (2)	0.00031 (16)	0.00196 (15)	0.00041 (15)
Cl1	0.0376 (3)	0.0546 (4)	0.0284 (3)	0.0153 (3)	0.0027 (2)	−0.0004 (2)
S1	0.0709 (4)	0.0438 (4)	0.0278 (3)	−0.0271 (3)	−0.0134 (3)	0.0079 (3)
N1	0.0322 (10)	0.0303 (10)	0.0371 (10)	−0.0001 (8)	0.0009 (8)	0.0009 (8)
N2	0.0310 (9)	0.0302 (10)	0.0261 (9)	−0.0020 (8)	0.0000 (7)	−0.0012 (7)
C1	0.0358 (12)	0.0290 (11)	0.0287 (11)	0.0006 (9)	−0.0023 (9)	0.0031 (9)
C2	0.0326 (11)	0.0336 (12)	0.0310 (11)	−0.0032 (9)	−0.0064 (9)	−0.0002 (9)
C3	0.0436 (13)	0.0287 (11)	0.0259 (10)	−0.0068 (9)	−0.0037 (9)	−0.0022 (9)
C4	0.0454 (14)	0.0302 (13)	0.0637 (17)	0.0008 (11)	−0.0125 (12)	0.0064 (12)
C5	0.0309 (12)	0.0374 (13)	0.0743 (18)	−0.0003 (10)	−0.0086 (12)	0.0023 (13)
C6	0.0339 (11)	0.0340 (11)	0.0265 (10)	−0.0025 (9)	0.0031 (9)	0.0002 (9)
C7	0.0345 (11)	0.0352 (12)	0.0333 (12)	−0.0093 (10)	−0.0004 (9)	0.0005 (10)
C8	0.0437 (13)	0.0265 (11)	0.0269 (11)	−0.0063 (9)	−0.0058 (9)	0.0022 (9)
C9	0.0495 (14)	0.0531 (15)	0.0280 (12)	−0.0121 (12)	0.0079 (10)	−0.0043 (11)
C10	0.0346 (12)	0.0550 (15)	0.0340 (12)	−0.0127 (11)	0.0061 (10)	−0.0060 (11)

### Geometric parameters (Å, °)

**Table:**

Co1—N1^i^	2.2184 (18)	C2—C3	1.379 (3)
Co1—N1	2.2185 (18)	C2—H2	0.9300
Co1—N2^ii^	2.2822 (17)	C3—C4	1.388 (3)
Co1—N2^iii^	2.2822 (17)	C4—C5	1.386 (3)
Co1—Cl1	2.4221 (5)	C4—H4	0.9300
Co1—Cl1^i^	2.4222 (5)	C5—H5	0.9300
S1—C8	1.766 (2)	C6—C7	1.376 (3)
S1—C3	1.775 (2)	C6—H6	0.9300
N1—C1	1.334 (3)	C7—C8	1.384 (3)
N1—C5	1.338 (3)	C7—H7	0.9300
N2—C10	1.340 (3)	C8—C9	1.385 (3)
N2—C6	1.340 (3)	C9—C10	1.381 (3)
N2—Co1^iv^	2.2822 (17)	C9—H9	0.9300
C1—C2	1.373 (3)	C10—H10	0.9300
C1—H1	0.9300		
			
N1^i^—Co1—N1	180.0	C1—C2—H2	120.5
N1^i^—Co1—N2^ii^	94.00 (6)	C3—C2—H2	120.5
N1—Co1—N2^ii^	86.00 (6)	C2—C3—C4	118.2 (2)
N1^i^—Co1—N2^iii^	86.00 (6)	C2—C3—S1	121.68 (17)
N1—Co1—N2^iii^	94.00 (6)	C4—C3—S1	119.80 (17)
N2^ii^—Co1—N2^iii^	180.00 (8)	C5—C4—C3	118.1 (2)
N1^i^—Co1—Cl1	89.50 (5)	C5—C4—H4	120.9
N1—Co1—Cl1	90.50 (5)	C3—C4—H4	120.9
N2^ii^—Co1—Cl1	90.03 (4)	N1—C5—C4	124.4 (2)
N2^iii^—Co1—Cl1	89.97 (4)	N1—C5—H5	117.8
N1^i^—Co1—Cl1^i^	90.50 (5)	C4—C5—H5	117.8
N1—Co1—Cl1^i^	89.50 (5)	N2—C6—C7	124.10 (19)
N2^ii^—Co1—Cl1^i^	89.97 (4)	N2—C6—H6	118.0
N2^iii^—Co1—Cl1^i^	90.03 (4)	C7—C6—H6	118.0
Cl1—Co1—Cl1^i^	179.999 (1)	C6—C7—C8	119.2 (2)
C8—S1—C3	102.90 (10)	C6—C7—H7	120.4
C1—N1—C5	115.77 (19)	C8—C7—H7	120.4
C1—N1—Co1	119.81 (14)	C7—C8—C9	117.7 (2)
C5—N1—Co1	124.16 (15)	C7—C8—S1	123.87 (17)
C10—N2—C6	115.96 (18)	C9—C8—S1	118.24 (16)
C10—N2—Co1^iv^	121.39 (14)	C10—C9—C8	119.1 (2)
C6—N2—Co1^iv^	122.47 (13)	C10—C9—H9	120.5
N1—C1—C2	124.4 (2)	C8—C9—H9	120.5
N1—C1—H1	117.8	N2—C10—C9	124.0 (2)
C2—C1—H1	117.8	N2—C10—H10	118.0
C1—C2—C3	119.0 (2)	C9—C10—H10	118.0
			
N2^ii^—Co1—N1—C1	−149.12 (16)	S1—C3—C4—C5	172.1 (2)
N2^iii^—Co1—N1—C1	30.88 (16)	C1—N1—C5—C4	3.4 (4)
Cl1—Co1—N1—C1	−59.12 (16)	Co1—N1—C5—C4	−170.7 (2)
Cl1^i^—Co1—N1—C1	120.88 (16)	C3—C4—C5—N1	−2.4 (4)
N2^ii^—Co1—N1—C5	24.8 (2)	C10—N2—C6—C7	−0.6 (3)
N2^iii^—Co1—N1—C5	−155.2 (2)	Co1^iv^—N2—C6—C7	−175.65 (16)
Cl1—Co1—N1—C5	114.8 (2)	N2—C6—C7—C8	0.6 (3)
Cl1^i^—Co1—N1—C5	−65.2 (2)	C6—C7—C8—C9	0.0 (3)
C5—N1—C1—C2	−1.0 (3)	C6—C7—C8—S1	−174.41 (17)
Co1—N1—C1—C2	173.43 (16)	C3—S1—C8—C7	−41.6 (2)
N1—C1—C2—C3	−2.4 (3)	C3—S1—C8—C9	144.02 (19)
C1—C2—C3—C4	3.3 (3)	C7—C8—C9—C10	−0.6 (4)
C1—C2—C3—S1	−169.79 (17)	S1—C8—C9—C10	174.20 (19)
C8—S1—C3—C2	−51.6 (2)	C6—N2—C10—C9	0.0 (4)
C8—S1—C3—C4	135.4 (2)	Co1^iv^—N2—C10—C9	175.13 (19)
C2—C3—C4—C5	−1.0 (4)	C8—C9—C10—N2	0.6 (4)

Symmetry codes: (i) −*x*, −*y*+1, −*z*+1; (ii) *x*−1, −*y*+3/2, *z*−1/2; (iii) −*x*+1, *y*−1/2, −*z*+3/2; (iv) −*x*+1, *y*+1/2, −*z*+3/2.

### Hydrogen-bond geometry (Å, °)

**Table:**

*D*—H···*A*	*D*—H	H···*A*	*D*···*A*	*D*—H···*A*
C5—H5···N2^ii^	0.93	2.62	3.119 (3)	114
C6—H6···Cl1^iv^	0.93	2.66	3.292 (2)	126
C10—H10···Cl1^v^	0.93	2.64	3.292 (2)	128

Symmetry codes: (ii) *x*−1, −*y*+3/2, *z*−1/2; (iv) −*x*+1, *y*+1/2, −*z*+3/2; (v) *x*+1, −*y*+3/2, *z*+1/2.
